# Understanding Amyotrophic Lateral Sclerosis: Pathophysiology, Diagnosis, and Therapeutic Advances

**DOI:** 10.3390/ijms25189966

**Published:** 2024-09-15

**Authors:** Radu Eugen Rizea, Antonio-Daniel Corlatescu, Horia Petre Costin, Adrian Dumitru, Alexandru Vlad Ciurea

**Affiliations:** 1 Department of Neurosurgery, University of Medicine and Pharmacy, “Carol Davila”, 020021 Bucharest, Romania; radu.rizea@umfcd.ro (R.E.R.); antonio.corlatescu0920@stud.umfcd.ro (A.-D.C.); horia-petre.costin0720@stud.umfcd.ro (H.P.C.); prof.avciurea@gmail.com (A.V.C.); 2 Department of Neurosurgery, “Bagdasar-Arseni” Clinical Emergency Hospital, 041915 Bucharest, Romania; 3 Department of Morphopathology, University of Medicine and Pharmacy, “Carol Davila”, 020021 Bucharest, Romania; 4 Emergency University Hospital Bucharest, 050098 Bucharest, Romania; 5 Sanador Clinical Hospital, 010991 Bucharest, Romania

**Keywords:** amyotrophic lateral sclerosis (ALS), therapeutic approaches, gene therapy, stem cell therapy, biomarkers, neurodegeneration

## Abstract

This review offers an in-depth examination of amyotrophic lateral sclerosis (ALS), addressing its epidemiology, pathophysiology, clinical presentation, diagnostic techniques, and current as well as emerging treatments. The purpose is to condense key findings and illustrate the complexity of ALS, which is shaped by both genetic and environmental influences. We reviewed the literature to discuss recent advancements in understanding molecular mechanisms such as protein misfolding, mitochondrial dysfunction, oxidative stress, and axonal transport defects, which are critical for identifying potential therapeutic targets. Significant progress has been made in refining diagnostic criteria and identifying biomarkers, leading to earlier and more precise diagnoses. Although current drug treatments provide some benefits, there is a clear need for more effective therapies. Emerging treatments, such as gene therapy and stem cell therapy, show potential in modifying disease progression and improving the quality of life for ALS patients. The review emphasizes the importance of continued research to address challenges such as disease variability and the limited effectiveness of existing treatments. Future research should concentrate on further exploring the molecular foundations of ALS and developing new therapeutic approaches. The implications for clinical practice include ensuring the accessibility of new treatments and that healthcare systems are equipped to support ongoing research and patient care.

## 1. Introduction

Amyotrophic lateral sclerosis (ALS) is a fatal neurodegenerative disease that impacts the central nervous system and poses significant challenges in diagnosis, particularly during its initial stages. Due to its rarity, physicians frequently prioritize more common diseases, resulting in diagnostic delays. Although the prevalence of ALS is low because of the short life expectancy of affected individuals, the lifetime risk of developing ALS is approximately 1 in 350 [1].

Identifying the diverse symptoms of ALS and recognizing it as a complex syndrome that often includes behavioral changes may aid in earlier diagnosis. Improvements in diagnostic criteria and the identification of genetic risk factors are also expected to accelerate the diagnostic process [2].

Diagnosing ALS remains a significant challenge, with little change in the diagnostic approach over the past decade. Despite the increased use of genetic testing, clinical history and examination are still the primary methods for confirming an ALS diagnosis. On average, it takes between 10 and 16 months from the onset of symptoms to receive a definitive diagnosis. This delay is attributed to the rarity and unfamiliarity of ALS, incomplete symptom recognition, and the lack of early specialist involvement [3]. Furthermore, the prognosis for ALS patients is poor, as the factors influencing disease progression are not fully understood.

To facilitate earlier diagnosis and improve outcomes, ongoing research is focusing on new ALS criteria and scoring systems, as well as the development of emerging biofluid markers, imaging techniques, and electrophysiological measurements. Recent advances in understanding the genotype-phenotype expression of familial amyotrophic lateral sclerosis (FALS) have aligned clinical, pathological, and genetic observations within families, revealing several presentation patterns predominantly associated with autosomal dominant (AD) inheritance [4]. The first pattern observed involves a rapid decline in motor function within five years, characterized by anterior horn cell (AHC) loss and corticospinal tract (CST) degeneration. A second pattern, clinically similar to the first, includes additional postmortem changes in the posterior columns, Clarke’s column, and spinocerebellar tracts. A third pattern, also similar to the second, features a longer survival period of over 10–20 years.

Patients with FALS associated with frontotemporal dementia (FTD) (FTD-ALS) exhibit multisystem manifestations, known as ALS-plus syndromes [5], which encompass cognitive and neurobehavioral impairments in addition to motor symptoms. These patients often experience bulbar-onset ALS and pathogenic gene expansions or mutations twice as frequently, resulting in generally shorter survival compared to those without ALS-plus syndromes. In rare cases, some FALS patients experience onset in their early teens, which is particularly devastating.

## 2. Epidemiology and Risk Factors

The incidence of ALS increases with age, reaching its peak between 60 and 79 years, though variations can occur based on ancestral background [6,7,8]. While some research suggests that incidence rates have remained stable over the past few decades [1], other studies indicate a potential increase [9,10]. These perceived changes might stem from improved diagnostic methods or evolving reporting standards, underscoring the importance of well-maintained population registries. Whether ALS incidence has significantly changed in the last twenty years remains uncertain, but it is expected to rise with the aging population [11]. Additionally, the prevalence of ALS is projected to increase due to an aging population and advancements in disease management that support longer life expectancy [11,12].

ALS incidence also varies by sex, with an overall standardized male-to-female ratio of 1.35, influenced by the age at onset [13]. Genetics is another contributing factor; heritability is higher in mother-daughter pairs [1], and the most common ALS risk gene, *C9ORF72*, results in an earlier onset in males compared to females [14]. Therefore, ALS development is driven by a complex interaction of age, sex, and genetic factors [15], which has significant implications for both preclinical and clinical research, as well as clinical trials.

ALS incidence is influenced by ancestral background and biological sex, with variations seen across different age groups [15]. Although the disease is more prevalent in males, heritability appears to be higher among females, particularly with the highest concordance observed in female–female parent–offspring pairs [1]. Additionally, males with the *C9ORF72* repeat expansion typically experience the onset of ALS approximately two years earlier than females with the same genetic marker [14]. Thus, the risk of developing ALS is shaped by a complex interplay of age, sex, and genetic factors [15]. These sex-based disparities underscore the importance of considering sex as a factor in both preclinical and clinical research to fully understand its impact, as well as in ALS clinical trials for the development of targeted treatments.

Amyotrophic lateral sclerosis (ALS) is influenced by both genetic and environmental factors. Familial ALS (FALS) accounts for 5–10% of cases, while the remaining 90–95% are sporadic ALS (SALS). Men are more likely to develop ALS than women, with a risk ratio ranging from 1.3 to 1.56 times higher. Various factors have been investigated to understand their impact on ALS risk, including smoking, physical activity, and exposure to metals, pesticides, and electromagnetic fields. Smoking has been consistently linked to an increased risk of ALS. However, the relationship between physical activity, particularly intense sports, and ALS remains unclear. Work-related exposure to metals such as lead and mercury has been proposed as a potential risk factor for ALS in several studies. Additionally, military service, especially during the Gulf War, has been associated with a higher incidence of ALS, possibly due to environmental exposures like high selenium levels from oil fires. Traumatic brain injuries and occupations involving electrical work have also been examined as risk factors, but the findings are mixed. These insights underscore the complex interplay between genetic predisposition and environmental factors in the development of ALS [16,17].

## 3. Pathophysiology

It is increasingly evident that a well-functioning protein degradation system is crucial for cellular health. In mammalian cells, protein degradation occurs via two primary pathways: the ubiquitin-proteasome system (UPS) and autophagy mechanisms. The UPS primarily degrades small, soluble proteins that are tagged for destruction, whereas autophagy handles the clearance of larger protein complexes. Although this suggests that enhancing autophagy might be an effective strategy for removing toxic *TDP*-*43* aggregates, the formation and progression of these aggregates beyond protein monomers may point to an initial dysfunction in the UPS. Furthermore, *TDP*-*43* inclusions are known to accumulate when both UPS and autophagy are inhibited [18,19,20,21].

Up to 10% of ALS patients have at least one other affected family member, classifying their condition as familial ALS (fALS), which is almost always inherited in an autosomal dominant manner [22]. Although over 50 potentially causative or disease-modifying genes have been identified, the most frequent pathogenic variants occur in *SOD1*, *C9ORF72*, *FUS*, and *TARDBP*, with disease-causing variants in other genes being relatively uncommon [23]. The prevalence of ALS cases attributed to variants in these common ALS-linked genes varies between European and Asian populations. Genetic risk factors are thought to significantly contribute to sporadic ALS (sALS), with twin studies estimating heritability at around 60% [24]. Despite numerous genetic association studies, identifying heritable genetic risk factors in sALS remains challenging.

The method of genetic diagnosis may also contribute to the apparent missing heritability in ALS. Genetic diagnosis is often conducted through whole exome sequencing, which can miss crucial intronic and intergenic variants. An increase in rare variants, many of unknown significance, has been found in the untranslated regions of known disease-causing genes, including *SOD1*, *TARDBP*, *FUS*, *VCP*, *OPTN*, and *UBQLN2*, highlighting the potential importance of regulatory gene regions in determining disease pathogenesis and making genetic diagnoses [25]. Heritability can also be difficult to determine due to the incomplete penetrance of variants in many ALS-associated genes. For example, *C9ORF72* and *ATXN2* variants exhibit incomplete penetrance, with symptoms not always manifesting in mutation carriers [14,26]. This can cause inherited cases to appear sporadic.

A substantial portion of the genetic risk for sALS remains unidentified; as a result, much research has focused on understanding how variations and differences in the expression of known ALS-linked genes lead to the disease. The genes *SOD1*, *TARDBP*, *FUS*, and *C9ORF72* have been the most extensively studied.

In the field of neurodegenerative diseases, including Alzheimer’s, Parkinson’s, and Huntington’s (AD-PD-HD), and ALS neuroinflammation has emerged as a crucial factor driving the progression of neuronal degeneration. Research has increasingly highlighted the significant role that chronic inflammation in the brain plays in exacerbating these conditions.

Multiple studies have shown that cannabinoids possess properties that can effectively reduce this neuroinflammatory burden. These compounds, derived from the cannabis plant, interact with the endocannabinoid system in the brain, which is involved in regulating inflammation and neuroprotection. By modulating immune responses and reducing inflammatory cytokines, cannabinoids have demonstrated potential in mitigating the harmful effects of neuroinflammation. This growing body of evidence suggests that cannabinoid-based therapies could offer promising new avenues for treating neurodegenerative diseases by addressing one of their key underlying mechanisms [27].

## 4. Protein Misfolding and Aggregation: Role of *C9ORF72, TDP-43, SOD1, FUS*

### 4.1. C9ORF72

The hexanucleotide repeat expansion is located in the first intron of the *C9ORF72* gene. The actual sizes of these repeats vary significantly among *C9ORF72* patients. Although the threshold for pathogenic repeat size remains unknown, a cutoff of 30 repeats is commonly used in most studies. Healthy individuals typically have fewer than 11 repeats, while most patients exhibit several hundreds to thousands of repeats [28]. A small subset of patients also display shorter expansions ranging from 45 to 80 repeats [29]. 

The repeat expansion in the *C9ORF72* gene is a major genetic factor in ALS and FTD, accounting for approximately 40% of familial ALS (FALS), 30% of familial FTD (FFTD), and about 8% of sporadic ALS (SALS) cases, particularly in predominantly Caucasian populations. The discovery of the *C9ORF72* mutation in ALS and FTD has led scientists to explore its potential involvement in other neurodegenerative disorders as well [30]. 

Regarding cell type distribution, *C9ORF72* expression is notably high in myeloid cells, particularly in CD14+ monocytes, eosinophils, and neutrophils, while it is lower in lymphoid cells and other tissues. Overall, the *C9ORF72* protein is mainly expressed in the brain, spinal cord, and immune system, with lower levels detected in other organs such as the lungs, heart, liver, kidney, and skeletal muscle, reflecting the transcript’s expression profile [31].

The *C9ORF72* gene transcribes three distinct mRNA isoforms (V1–V3), which encode two proteins: *C9ORF72*-long (C9-L), a 481-amino acid protein, and *C9ORF72*-short (C9-S), a truncated protein isoform of 222 amino acids [32]. 

The 481-amino acid C9-L isoform is the most abundant. In mouse tissues and the human brain, *C9ORF72* is predominantly cytoplasmic, with punctate staining in neurites, indicative of synaptic terminals. *C9ORF72* also plays a role in stress granule (SG) formation and degradation [33]. Stress granules (SGs) are membraneless ribonucleoproteins that contain mRNA and accumulate in the cytoplasm when translation initiation is halted, disappearing once stress is alleviated [34]. Upon stress-related stimuli, C9-L is recruited to SGs. In the absence of *C9ORF72*, these granules fail to form, and other SG-associated proteins like TIA-1, G3BP1, and HuR are downregulated. *C9ORF72* is also crucial for cellular recovery post-stress, as it interacts with p62 to target SGs for autophagy-mediated degradation [35]. 

Moreover, C9-L interacts with *SMCR8* through its DENN (differentially expressed in normal cells and neoplasia) domain, and when the C9-L-SMCR8 complex associates with *WDR41*, it functions as a GTPase-activating protein potentially involved in membrane trafficking, including endolysosomal pathways and autophagy [36]. The loss of *C9ORF72* in mice and motor neurons derived from iPSCs leads to synaptic plasticity alterations and defects in glutamate receptor homeostasis [37]. Recent studies have also implicated *C9ORF72* in regulating neuronal nucleocytoplasmic transport by disrupting interactions between importin β1 and nucleoporin [38]. 

According to a study conducted by Celona et al., the expansion of a six-nucleotide GGGGCC repeat in the first intron of the *C9ORF72* gene is the most prevalent inherited cause of amyotrophic lateral sclerosis (ALS) and frontotemporal dementia (FTD). These repeated sequences in RNA can aggregate within cells and are translated through a mechanism called repeat-associated non-AUG (RAN) translation, producing dipeptide-repeat (DPR) proteins that are highly toxic. The study reveals that the zinc finger RNA-binding protein Zfp106 plays a critical role in counteracting these harmful effects. Zfp106 was shown to prevent the formation of RNA foci, substantially decrease RAN translation driven by GGGGCC repeats, and inhibit the accumulation of toxic DPRs in cells derived from *C9ORF72* patients. Additionally, the study found that Zfp106 binds to RNA G-quadruplex structures, inducing a change in their conformation. This binding appears to be a key factor in *Zfp106*’s ability to mitigate the cytotoxic effects associated with GGGGCC repeats, highlighting its potential as a therapeutic target for *C9ORF72*-linked ALS and FTD [39].

There is widespread agreement that the arginine-rich dipeptide repeats (DPRs), specifically glycine-arginine (GR) and proline-arginine (PR), exhibit the highest neurotoxicity across multiple model systems [40]. Due to the growing interest in these arginine-rich DPRs, there has been a greater focus on PR, GR, or both, compared to other DPRs. Consequently, several mechanisms have been associated with PR and GR expression, including nucleocytoplasmic transport [41,42], DNA damage [43,44], translational disruption [45], and stress granule dysfunction [46]. Drosophila models have been particularly valuable in elucidating these toxic mechanisms, which have subsequently been validated in mouse models, induced pluripotent stem cells (iPSCs), and patient tissues.

Recent advancements with fly models continue to prioritize research on repeat-associated non-AUG (RAN) translation and DPRs, given their role in *C9orf72* hexanucleotide repeat expansions. Factors regulating RAN translation are under investigation, with a proposed mechanism of DPR-induced toxicity in *C9orf72*-associated neurodegeneration involving inhibited translation through interactions between ribosomal proteins and DPRs, specifically poly-GR and poly-PR.

Emerging evidence indicates that the three primary hypotheses regarding DPR toxicity are not mutually exclusive. For example, one study demonstrated that reduced *C9orf72* activity heightens susceptibility to degenerative stimuli, such as glutamate-induced excitotoxicity and impaired DPR clearance [47]. Essentially, haploinsufficiency intensifies toxic gain-of-function effects, a finding supported by studies showing that *C9orf72* reduction suppresses autophagy, leading to DPR accumulation and neuronal death [48,49].

Research on autophagy in Drosophila *C9orf72* models has shown that, in motor neurons, 30-repeat DPRs disrupt the morphology and dynamics of the endoplasmic reticulum (ER), impairing autophagosome formation. Despite ER disruption in both axons and synapses, autophagosomes remained intact in axons, although their biogenesis was hindered at synaptic terminals [50].

Motor neurons can also be analyzed for synaptic dysfunction. A recent study using Drosophila *C9orf72* revealed a novel cell-autonomous excitotoxicity mechanism selectively associated with arginine-rich DPRs in glutamatergic neurons. These DPRs—poly-GR and poly-PR—exhibited moderate toxicity at 36 repeats, increasing synaptic boutons, active zones, extracellular glutamate, intracellular calcium, and presynaptic NMDA receptor activation [51]. This suggests a pathway to neurodegeneration via glutamate excitotoxicity and synaptic overgrowth, which was presynaptic NMDA receptor-dependent and, therefore, cell-autonomous. More toxic 100-repeat DPRs led to the loss of active zones, indicating severe neurodegeneration through synaptic degeneration [51]. These findings support the potential of glutamate inhibition therapies and underscore the need for further investigation into synaptic dysfunction in *C9orf72*-FTD pathology.

### 4.2. TDP-43

TAR DNA-binding protein 43 (*TDP-43*) is a vital RNA/DNA-binding protein involved in various aspects of RNA metabolism, including transcription, splicing, and transport. In amyotrophic lateral sclerosis (ALS), *TDP*-*43* is primarily associated with its pathological forms. In ALS, *TDP*-*43* mislocalizes from the nucleus to the cytoplasm, where it forms insoluble and ubiquitinated inclusions. These pathological aggregates are a hallmark of both ALS and frontotemporal lobar degeneration (FTLD). The misfolding and aggregation of *TDP-43* are driven by mutations in the *TARDBP* gene, post-translational modifications such as phosphorylation and ubiquitination, and its tendency to form amyloid-like fibrils. This mislocalization disrupts RNA processing and cellular homeostasis, leading to neuronal toxicity. Understanding the mechanisms underlying *TDP*-*43* pathology is crucial for developing targeted therapies for ALS [52,53]. 

A proposed model for *TDP-43* toxicity utilizing Drosophila suggests that the cytoplasmic accumulation of human *TDP-43* (*hTDP-43*) in adult flies is sufficient to induce degeneration. This finding aligns with other models of toxicity related to the mislocalization of ALS-associated genes. Additionally, the study observed that the knockdown of *TBPH*, the Drosophila homolog of *TDP-43*, did not produce any phenotypic changes, indicating that a toxic gain of function due to cytoplasmic *TDP-43* may contribute to pathogenesis. However, this hypothesis remains uncertain, as a loss of nuclear *TDP-43* function might also play a role in the development of the disease [54].

Another proposed model of toxicity in Drosophila involves mitochondrial dysfunction. In transgenic flies expressing human *TDP-43* (*hTDP-43*) induced by heat shock through the Elav-Gal4 pan-neuronal driver, the mitochondria in the eye exhibited a marked reduction in size compared to controls. Additionally, 85% of the mitochondria in the photoreceptors of *hTDP-43*-expressing flies displayed swollen or vesicular cristae. This damage to the mitochondrial cristae mirrors that observed in the brain tissues of patients with *hTDP-43* proteinopathy, underscoring the utility of Drosophila as a model for studying this gene. Given that mitochondria are a known source of reactive oxygen species (ROS), mitochondrial dysfunction can lead to ROS accumulation, negatively impacting neuronal survival and function. Elevated levels of mitochondrial ROS were detected in *hTDP-43*-expressing motor neurons in Drosophila via confocal imaging, suggesting that *hTDP-43* expression in motor neurons induces mitochondrial dysfunction and may contribute to oxidative stress [55].

### 4.3. SOD1

The *SOD1* gene (encoding superoxide dismutase 1 [Cu/Zn]) was the first to be linked to ALS in 1993 [56]. *SOD1* encodes a metalloenzyme composed of 153 amino acids and is one of three superoxide dismutase enzymes in humans. This protein binds copper and zinc and forms a highly stable homodimer. *SOD1* dimers are located in the cytosol and the intermembrane space of mitochondria, playing a crucial role in antioxidant defense by catalyzing the conversion of superoxide radicals, produced during cellular respiration, into oxygen and hydrogen peroxide [57].

Superoxide dismutase 1 (*SOD1*) is crucial in the pathogenesis of amyotrophic lateral sclerosis (ALS). Mutations in the *SOD1* gene are a primary genetic cause of familial ALS, accounting for approximately 20% of these cases. These mutations result in the misfolding and aggregation of the *SOD1* protein, which is highly toxic to motor neurons. The toxic effects are believed to arise from a combination of factors, including oxidative stress, mitochondrial dysfunction, and disruption of protein homeostasis. Mutant *SOD1* proteins can form aggregates that interfere with cellular processes, disrupt mitochondrial function by localizing to the outer mitochondrial membrane, and induce apoptosis. Additionally, the presence of misfolded *SOD1* has been shown to enhance the aggregation of other ALS-associated proteins, such as *TDP*-*43*, further amplifying the neurodegenerative process. Understanding *SOD1’s* role in ALS has been pivotal for developing targeted therapies aimed at reducing oxidative stress and improving mitochondrial function to slow disease progression [58].

Drosophila melanogaster models have been pivotal in elucidating the role of the *SOD1* gene in amyotrophic lateral sclerosis (ALS). As the first gene linked to familial ALS (fALS), *SOD1* encodes the antioxidant enzyme superoxide dismutase-1. By leveraging genetic tools to express human *SOD1* (*hSOD1*) and its ALS-associated mutations in specific Drosophila tissues, researchers have uncovered key insights into the disease’s mechanisms. These models demonstrate that *hSOD1* mutations can trigger both loss of function (LOF) and toxic gain of function (GOF) effects, leading to neurodegeneration characterized by motor dysfunction, reduced lifespan, and synaptic abnormalities. Notably, despite severe phenotypes such as developmental defects and impaired locomotion, these models often lack significant neuronal death, differing from vertebrate observations. This highlights the complexity of *SOD1*-linked ALS and underscores the value of Drosophila in studying the disease’s molecular and cellular underpinnings. Additionally, these models have been instrumental in screening potential therapeutic compounds, identifying agents that may alleviate the neurotoxic effects of mutant *SOD1* [59].

Moreover, a study conducted by Liguori et al. (2024), utilizing Drosophila models expressing the human mutant *SOD1* genes (A4V and G85R), demonstrated that these mutations lead to reduced survival and impaired motor performance, concurrently with the induction of early neuroinflammatory markers, such as the glial marker Repo, and the upregulation of immune pathways involving antimicrobial peptides (AMPs). Moreover, the study shed light on the heightened oxidative stress and the presence of chromosome aberrations, indicating that genomic instability and altered cellular homeostasis represent some of the most important moments in the early stages of ALS pathogenesis. These findings challenge the prevailing notion that motor neuron degeneration is the primary initiating factor in ALS, instead emphasizing the possibility that early neuroinflammation and oxidative stress may precede and potentially precipitate the cascade of neuronal damage, thereby suggesting new opportunities for early therapeutic intervention [60].

Furthermore, a study conducted by Scaricamazza et al [61]. showed that endurance exercise in female mice can lead to the aggravation of the *SOD1*-G93A ALS onset since it induces a more oxidative phenotype in muscle fibers, exacerbating muscle denervation, and promoting motor neuron loss. Additionally, due to increased immune cell infiltration in the sciatic nerve, the progression of ALS is accelerated. Therefore, despite the fact that physical exercises are a healthy habit, endurance exercises represent risk factors when it comes to ALS [61]. 

### 4.4. FUS

*FUS* encodes a universally expressed protein of 526 amino acids, part of the FET family of RNA-binding proteins. Under normal conditions, *FUS* is mainly located in the nucleus but can move to the cytoplasm, where it plays a role in nucleocytoplasmic transport [62,63]. *FUS* has several roles similar to *TDP*-*43*, including involvement in gene expression, transcription, pre-mRNA splicing, RNA transport, and regulation of translation [64]. However, despite these similarities, *TDP*-*43* and *FUS* target different RNAs and have unique sequence binding specificities [65].

As of now, researchers have identified more than 50 distinct variants of the *FUS* gene that are linked to autosomal dominant forms of ALS. The majority of these genetic alterations are missense mutations, which involve single amino acid changes. However, there have also been less common cases where insertions, deletions, splicing errors, or nonsense mutations have been observed in patients. These various mutations contribute to the diversity of genetic presentations seen in ALS associated with *FUS* gene anomalies [66]. Many pathogenic variants are located within the nuclear localization signal, leading to the mislocalization of *FUS* to the cytoplasm. Other mutations occur in regions rich in glycine and arginine, the prion-like domain, and the 3′ untranslated region (3′UTR) [67]. Some variants in these regions increase the likelihood of the protein forming solid aggregates, indicating multiple pathogenic mechanisms in *FUS*-related ALS [68].

RNA homeostasis, or “ribostasis”, is as essential as proteostasis. Disruptions in RNA metabolism can cause toxicity in various ways, some of which are linked to proteostasis. As per the central dogma of molecular biology, RNA acts as the “messenger” between DNA and the protein synthesis machinery, implicating RNA in the pathogenicity of protein misfolding diseases like ALS. However, RNA’s lifecycle is complex, involving numerous regulatory RNA-binding proteins (RBPs) that play crucial roles in RNA transport, posttranscriptional editing, translation, and degradation [69]. Furthermore, RNA and RBPs can form membraneless organelles called ribonuclear protein (RNP) granules, which are vital for RNA metabolism and gene regulation [70]. The functions of these RNP granules vary depending on the specific RNA and RBPs they contain. Recent studies have focused on particular RNPs, such as stress granules and paraspeckles, both of which have been implicated in ALS pathogenesis. The role of stress granules, in particular, has been debated as a key factor in ALS-related aggregation. However, since ALS can develop independently of stress granules, they will not be further examined here as a therapeutic target, though they have been reviewed in other works [71,72,73,74].

The regulation and maintenance of RNA pathways via RBPs and RNP granules are crucial for cellular homeostasis. Notably, several strong genetic links to ALS have been found in RNA metabolism pathways, including mutations in *C9ORF72*, *SOD1*, *TDP*-*43*, and *FUS*. These mutations and others underscore the importance of RNA production, editing, and localization in the onset of ALS, providing convincing evidence that deregulated RNA metabolism plays a significant role in the disease. The following sections will emphasize the significance of RBPs and RNP granule formation in ALS and their potential as therapeutic targets to restore proper RNA metabolism and proteostasis in ALS patients [75,76] (Table 1).

To elucidate the functions of *FUS*, research has concentrated on the role of the *cabeza* (*caz*) gene, which is the sole ortholog of *FUS* in Drosophila [77]. The *caz1* mutants were found to be morphologically normal but exhibited a defect in adult eclosion. The resulting adult escapers demonstrated a reduced lifespan and impaired locomotion [78]. Overexpression of *FUS* in the nervous system of *caz1* mutants in Drosophila was able to rescue the eclosion defect and restore both lifespan and locomotor function. However, the expression of mutated forms of *FUS* (P525L and R522G) affected the survival of *caz1* mutants to adulthood without influencing lifespan or locomotion in adults [78].

RNA interference (RNAi) lines used to knock down *caz* gene expression in neurons showed that *caz* silencing did not impact lifespan but did impair climbing performance [79]. Knockdown of *caz* in the eye led to a rough eye phenotype resulting from apoptotic cells in the pupal retina; this phenotype could be rescued by the expression of the anti-apoptotic protein p35 [80]. The generation of new *caz* null mutants and conditional alleles using homologous recombination confirmed the previously observed phenotypes of pupal lethality and locomotor defects [81]. These findings support the essential role of *caz* in neural development and provide evidence in favor of the loss-of-function (LOF) hypothesis to explain *FUS*-induced neurodegeneration in amyotrophic lateral sclerosis (ALS). Gain-of-function (GOF) of *caz* resulted in phenotypes similar to those observed with *FUS* overexpression, including impaired larval locomotion, reduced synaptic bouton number, and severe eye degeneration [82]. Additionally, both *caz* and *FUS* GOF induced apoptosis when expressed in motorneurons [79].

These findings further support (i) the concept of evolutionary conservation between *caz* and *FUS* and (ii) the pivotal role of *FUS* expression levels in triggering the neurodegenerative process [78,83,84]. Therefore, a deeper understanding of the physiological functions of *FUS* and its various interacting partners is crucial for elucidating the mechanisms involved in this pathology.

## 5. Clinical Presentation and Diagnosis

### 5.1. Symptoms: Initial Signs and Progression of ALS Symptoms

ALS presents with a diverse range of clinical features. The key characteristic is the coexistence of upper and lower motor neuron signs and symptoms. Upper motor neuron (UMN) findings include hyperreflexia, poor dexterity, incoordination, and spasticity. Common bulbar UMN findings are dysarthria and dysphagia. Lower motor neuron (LMN) findings include muscle atrophy and fasciculations [85]. Weakness, a central feature, can result from dysfunction in either upper or lower motor neurons. The initial presentation influences the pattern of symptom progression and has prognostic significance. Most patients initially present with asymmetric LMN symptoms localized to the arm or leg [86]. Common early clinical signs include hand weakness, shoulder girdle weakness, and foot drop. Bulbar-onset ALS, which occurs in 25% of patients, typically presents with dysarthria and dysphagia. A minority of patients initially present with respiratory muscle weakness or generalized weakness combined with bulbar muscle weakness [87].

In patients who develop cognitive symptoms, impaired word fluency is an early finding. Other features of executive dysfunction, such as mental inflexibility, inattention, disinhibition, or difficulty in planning or problem-solving, can emerge as the disease progresses [88]. Impairment in multiple cognitive domains is less common in ALS and, when present, usually involves language or memory and may be confounded by co-pathologies such as Alzheimer’s disease [89].

Behavioral abnormalities are a frequent neuropsychiatric feature in ALS, with apathy being the most common. Other behavioral changes include disinhibition, perseverative behavior, altered food preferences, loss of empathy, and impaired social cognition, including emotional processing [90,91]. Pathological crying and laughing, also known as emotional lability or pseudobulbar affect, is present in approximately one-third of patients with ALS and is associated with gray and white matter pathology in the cortico-cerebellar network. Pathological crying and laughing do not correlate with neuropsychological measures and should be distinguished from other cognitive and behavioral symptoms [92,93,94].

### 5.2. Diagnostic Criteria: Revised El Escorial Criteria and Other Diagnostic Tools

Diagnosing ALS is based on the El Escorial criteria. According to these criteria, diagnosis requires a history of progressive weakness spreading within a region or to other regions, such as the bulbar region (affecting speech and swallowing), cervical region (affecting the upper limbs), thoracic region (affecting the chest wall and abdominal muscles), or lumbar region (affecting the lower limbs). There must be evidence of lower motor neuron involvement (indicated by specific symptoms or denervation on electromyography) and upper motor neuron involvement (indicated by specific symptoms and brisk deep tendon reflexes) [95].

The development of the Awaji criteria, which stemmed from a revised algorithm for the clinical neurophysiological assessment in diagnosing ALS, formulated through a consensus among experts and grounded in the existing literature, has been criticized for potentially possessing several limitations [96].

The Awaji criteria proposed that neurophysiological indicators of lower motor neuron (LMN) dysfunction should be considered equivalent to clinical signs of LMN involvement, while the assessment of upper motor neuron (UMN) dysfunction remained reliant on clinical evaluation. Compared to the revised El Escorial criteria (rEEC), the Awaji criteria demonstrated increased sensitivity in several studies [97,98]. However, some studies reported lower sensitivities [99,100], a discrepancy attributed to the exclusion of the probable-laboratory-supported diagnostic category. Notably, the diagnostic advantage of the Awaji criteria was found to be most significant in cases of bulbar-onset ALS [96].

The use of the Awaji algorithm eliminates the somewhat artificial distinction between clinically definite ALS and EMG definite ALS, as it integrates the clinical and electrophysiological diagnostic processes, treating them as complementary rather than independent assessments [98].

In September 2019, an assembly of international neurologists convened in Gold Coast, Australia, with the objective of deconstructing and simplifying the diagnostic process for amyotrophic lateral sclerosis (ALS). The resulting Gold Coast criteria for ALS diagnosis require evidence of progressive motor impairment, which must be documented through patient history or repeated clinical assessments, following an initial period of normal motor function. Additionally, there must be evidence of both upper and lower motor neuron dysfunction within at least one body region. If dysfunction is confined to a single region, both upper and lower motor neuron impairments must be present in that region. In cases involving multiple regions, lower motor neuron dysfunction must be evident in at least two regions. Furthermore, the exclusion of other disease processes must be achieved through appropriate investigations tailored to the clinical presentation [101].

The criteria specify that upper motor neuron dysfunction is characterized by at least one of the following: increased deep tendon reflexes, the presence of pathological reflexes (such as the Hoffman or Babinski signs), increased muscle tone (spasticity), or slowed and poorly coordinated voluntary movements not attributable to lower motor neuron weakness or Parkinsonian features. In contrast, lower motor neuron dysfunction requires either clinical evidence of muscle weakness and wasting or electromyography (EMG) abnormalities. These EMG findings must indicate chronic neurogenic changes, such as large motor unit potentials, and evidence of ongoing denervation, which may manifest as fibrillation potentials or positive sharp waves [102].

The body regions relevant to the diagnosis are defined as bulbar, cervical, thoracic, and lumbosacral. A region is classified as involved if there are abnormalities in two limb muscles innervated by different roots and nerves or if there are abnormalities in a single bulbar or thoracic muscle. Appropriate diagnostic investigations may include nerve conduction studies, needle EMG, imaging techniques (such as magnetic resonance imaging), biofluid analysis, or other modalities deemed clinically necessary [101].

Amyotrophic Lateral Sclerosis (ALS) diagnosis is classified into four categories based on specific criteria. Definite ALS is identified by the presence of upper motor neuron (UMN) and lower motor neuron (LMN) signs in three anatomical regions. Probable ALS is diagnosed when UMN and LMN signs are present in at least two regions, with UMN signs rostral to LMN signs. Probable ALS, laboratory-supported, involves the presence of UMN and LMN signs in one region with electromyographic (EMG) evidence of LMN involvement in another region. Lastly, Possible ALS is characterized by the presence of UMN and LMN signs in one region or UMN signs in two or three regions, which may include conditions such as monomelic ALS, progressive bulbar palsy, and primary lateral sclerosis.

There is an average delay of 10–16 months from the onset of a patient’s symptoms to the confirmation of the diagnosis. The absence of a definitive biological marker for ALS, the highly variable initial clinical presentations of the disease, and its pathogenic overlap with several neurodegenerative disorders all contribute to the difficulty in diagnosing ALS with acceptable certainty [103].

Moreover, we should also take into account the importance of ALS staging. Two of the most important staging systems are represented by MiTo and King’s [104,105].

The King’s Clinical Staging System and the Milano-Torino (MiToS) Functional Staging System represent two distinct approaches for assessing the progression of amyotrophic lateral sclerosis (ALS). The King’s staging system divides ALS into five stages, which are determined by the extent of clinical involvement and its impact on feeding or respiratory function. Stage 1 denotes the onset of symptoms, while Stage 5 corresponds to death. Although the King’s staging is not directly derived from the ALS Functional Rating Scale-Revised (ALSFRS-R), there is a 92% concordance when estimating stages from ALSFRS-R scores. This system is primarily concerned with the anatomical spread of the disease and significant respiratory muscle involvement, providing greater specificity in distinguishing stages during the early to mid-phases of ALS.

Conversely, the MiToS staging system comprises six stages, from 0 to 5, based on functional abilities as measured by the ALSFRS-R. Stage 0 reflects normal function, whereas Stage 5 represents death. The MiToS system is specifically designed to evaluate the functional capabilities affected by ALS, with a particular focus on the later stages of the disease due to its emphasis on the functional burden.

While these two staging systems differ in their approaches, they are complementary rather than redundant, each offering unique perspectives on disease progression. The King’s system provides a more detailed resolution of disease spread in the early to mid-stages, emphasizing clinical and anatomical progression, while the MiToS system offers a more nuanced evaluation of functional impairment in the later stages. Therefore, employing both staging systems together is recommended to achieve a more comprehensive understanding of ALS progression [104,105].

Moreover, recent advancements in neuroimaging, including advanced computational methods and diffusion tensor imaging (DTI), have significantly improved the capacity to detect subtle alterations in brain structure and function. These technological developments enable a more detailed characterization of gray and white matter damage in patients, thereby facilitating the distinction between various subtypes of amyotrophic lateral sclerosis (ALS) and monitoring disease progression. For instance, novel MRI techniques have demonstrated the potential to differentiate between faster and slower-progressing ALS cases by analyzing patterns of cortical thinning, white matter integrity, and specific neuroanatomical changes associated with distinct clinical phenotypes [106].

### 5.3. Differential Diagnosis: Distinguishing ALS from Other Motor Neuron Diseases and Conditions

For patients with predominant upper motor neuron (UMN) involvement, conditions such as cervical radiculomyelopathy, hereditary spastic paraplegia, adrenomyeloneuropathy, and cerebrotendinous xanthomatosis should be considered. If only lower motor neuron (LMN) features are present, diagnoses like plexopathy, peripheral neuropathy (e.g., multifocal motor neuropathy with conduction block, chronic inflammatory demyelinating polyneuropathy, infectious neuropathy), or myopathies (e.g., inclusion body myositis) should be ruled out. Flail arm ALS needs to be distinguished from conditions such as spinal muscular atrophy, Kennedy’s disease, multifocal motor neuropathy, and monomelic amyotrophy. In cases of focal onset neck extensor weakness, myasthenia gravis and focal myopathy should be considered. Muscle-specific tyrosine kinase (MuSK) myasthenia can present with tongue weakness and atrophy, potentially being mistaken for bulbar ALS [107].

## 6. Current Therapeutic Approaches

For many years, Riluzole (6-(trifluoromethoxy)-2-aminobenzothiazole) was the only treatment available for ALS patients. The primary goal of this treatment was to reduce excitotoxicity, particularly from glutamate. The beneficial effects of Riluzole are believed to come from several actions. Firstly, it acts as a sodium channel blocker on presynaptic neurons, which decreases the release of glutamate into the synaptic cleft. Additionally, it increases glutamate reuptake by activating astrocytic excitatory amino acid transporter 2 (EAAT2) channels [108,109]. It also noncompetitively inhibits AMPA and N-methyl-D-aspartate (NMDA) glutamate receptors on postsynaptic neurons. Finally, Riluzole appears to reduce gamma-aminobutyric acid (GABA) reuptake and enhance GABA receptors [110].

Edaravone, the newest FDA-approved medication for ALS, acts as a powerful antioxidant and free radical scavenger. While its exact mechanism in treating ALS is not completely understood, its antioxidant properties are deemed crucial because oxidative stress significantly contributes to the disease’s progression [111].

Sodium phenylbutyrate/taurursodiol is another approved treatment for ALS, working by mitigating cellular stress pathways and promoting nerve cell survival. Patients taking this medication have shown a slower decline in daily functioning and increased survival rates compared to those on a placebo [112].

Tofersen, an RNA-based therapy, is approved to reduce the production of the superoxide dismutase type 1 (*SOD1*) protein by degrading toxic aggregates of *SOD1* mRNA (superoxide dismutase type-1 messenger ribonucleic acid). Starting Tofersen treatment has demonstrated reductions in neurofilament-axonal (nerve) injury, decreased neurodegeneration, and improved disease outcomes [113,114].

A study identified the combination of nebivolol and donepezil (nebivolol-donepezil) as a potential treatment for ALS by examining genetic information from ALS patients and pharmacogenomic data from the drug. The findings revealed that nebivolol-donepezil significantly reduced cytokine levels in microglial cell lines, inhibited nuclear factor-κB nuclear translocation in HeLa cells, protected against excitotoxicity-induced neuronal loss by modulating the PI3K-Akt pathway, and promoted the differentiation of neural precursor cells into motor neurons [115].

PXT864, a low-dose combination of aminocaproate and baclofen, has shown the ability to protect neuromuscular junctions and maintain motor neuron integrity in glutamatergic-injured primary neuron-muscle models, suggesting it could be a promising therapeutic strategy for ALS [116]. These findings indicate that combination therapies may help slow the progression of ALS, improve patients’ quality of life, and extend their life expectancy, making them highly promising treatment options.

Another therapeutic strategy that has gained renewed interest involves targeting muscle abnormalities in ALS. One rationale is neuroprotective, suggesting that changes in muscle and neuromuscular junctions may contribute to retrograde degeneration, as indicated by recent research on the role of muscle-secreted toxic exosomes in motor neuron degeneration [117]. The second approach focuses on symptom relief by increasing muscle contractility. This includes the development of two troponin activators, tirasemtiv [118,119] and reldesemtiv (NCT04944784), aimed at enhancing muscle contractility. Additionally, there are efforts to improve muscle mass and strength [120].

For frontotemporal dementia (FTD) or amyotrophic lateral sclerosis (ALS) associated with the C9orf72 expansion, structural and functional MRI studies indicate that subcortical structures, particularly the thalamus, are more likely to be affected compared to sporadic cases. Patients with FTD resulting from GRN or TBK1 mutations have been reported to exhibit prominently asymmetric atrophy, which is most noticeable in the later stages of the disease. In comparison to sporadic ALS, C9orf72-associated ALS demonstrates more extensive frontotemporal involvement, even in the absence of cognitive or behavioral symptoms. Additionally, many SOD1 variants are linked to more pronounced cervical spinal cord atrophy, with a relative preservation of cortical motor networks [121].

## 7. Emerging Therapies and Future Directions

### 7.1. Gene Therapy: Advances in Gene Editing and Antisense Oligonucleotides

Gene therapy for ALS is a promising approach that targets genetic mutations in *SOD1*, *C9ORF72*, *TARDBP*, and *FUS*. Various strategies have been developed to address these genetic abnormalities, including antisense oligonucleotides (ASOs) and RNA interference (RNAi) to degrade or inhibit abnormal mRNA, as well as gene delivery via viral vectors to replace defective genes. For example, ASOs targeting the *SOD1* gene have shown effectiveness in reducing toxic protein levels in preclinical studies and have advanced to clinical trials. Tofersen, an ASO against *SOD1* mRNA, demonstrated safety and tolerability in phase 1 trials, with phase 3 trials currently underway to confirm its ability to slow disease progression. Similarly, CRISPR/Cas9 technology has been used to correct mutations in genes like *FUS*, showing potential in preclinical models to restore normal function and reduce disease symptoms. These advancements underscore the significant potential of gene therapy to alter the course of ALS, though further research and clinical trials are needed to ensure long-term efficacy and safety [85,122,123].

### 7.2. Stem Cell Therapy: Potential of Stem Cell Treatments in ALS

Astrocytes and microglia play essential roles in ALS pathogenesis by promoting neuroinflammation. In the brain and spinal cord, these cells interact with neurons and with each other, influencing disease progression. Human induced pluripotent stem cells (hiPSCs) offer a valuable platform for studying ALS, providing insights into disease mechanisms across all genotypes and aiding in modeling sporadic cases. hiPSCs enable the development of high-throughput genetic and chemical screens [124]. Protocols for differentiating hiPSCs into astrocytes have been established for over a decade, while protocols for hiPSC-derived microglia have emerged more recently [125]. These protocols aim to mimic microglial development, distinct from blood macrophages, as microglia originate from yolk sac-derived progenitors [126,127]. Despite their potential, challenges remain in modeling ALS with hiPSCs. Simplified hiPSC-derived monocultures allow the study of cell-autonomous effects, but understanding non-cell-autonomous disease mechanisms requires modeling interactions between neurons and glia. Co-cultures of hiPSC-derived neurons and glia support glial maturation and have been used to study ALS patient-derived cells [128]. More complex in vitro models incorporating multiple cell types would be ideal.

For example, ezogabine, a potassium channel activator, reduced neuronal excitability in *SOD1* and *C9ORF72* hiPSC models, leading to a phase 2 clinical trial that showed decreased motor neuron excitability [129]. Similarly, the src/c-Abl inhibitor bosutinib improved hiPSC-derived motor neuron survival and muscle contractions in ALS models and is currently in clinical trials. Ropinirole, a dopamine agonist, showed positive effects in hiPSC-derived motor neurons from patients with *TARDBP*, *FUS* mutations, and sporadic ALS, leading to a phase I/IIa clinical trial [130]. Stem cell therapy holds promise for ALS treatment. hiPSCs allow the study of disease-specific mutations and their effects on neural cells, aiding in understanding ALS pathogenesis and testing therapies. Advances in gene editing, like CRISPR/Cas9, have furthered these studies. Despite challenges in achieving full cell maturation, hiPSC-based models continue to provide valuable insights and hold potential for developing effective ALS treatments.

## 8. Biomarkers for Early Detection and Progression: Recent Developments in Biomarker Research

### 8.1. Neurofilaments

Neurofilaments are among the most widely characterized fluid biomarkers in neurodegenerative diseases. Found only in neurons, these intermediate, filamentous proteins form part of the cytoskeletal structure and are particularly abundant in myelinated axons [131]. Released into the interstitial fluid during neuroaxonal injury and easily measured in cerebrospinal fluid (CSF), neurofilaments especially neurofilament light (NfL) and phosphorylated neurofilament heavy (pNfH) have become markers of neuronal injury and degeneration. Consequently, neurofilaments in CSF have been extensively studied as diagnostic, prognostic, susceptibility/risk, and response biomarkers for ALS.

Studies have shown that CSF and blood neurofilament levels are higher in ALS patients compared to those with ALS mimics, indicating their usefulness in distinguishing between the two groups [132,133]. This evidence suggests that fluid neurofilament concentrations could lead to a quicker and more accurate ALS diagnosis. However, adding other biomarkers involved in ALS pathophysiology and ALS mimic syndromes may further enhance diagnostic accuracy.

### 8.2. Chitinase

Non-neuronal cells play a role in ALS pathophysiology, with activated microglia and astrocytes causing chitinase expression. According to Thompson et al. [134], chitotriosidase-1 (CHIT1) and chitinase-3-like protein 2 (CHI3L2/YKL39) could differentiate ALS from mimicking conditions, but their diagnostic performance was weaker than neurofilament. CHIT1 and CHI3L2 were correlated with the rate of disease progression, and CHIT1 was correlated with survival when included in a multivariate model [134]. Another study conducted by Gille et al. [135] found that CHIT1 and chitinase-3-like protein 1 (CHI3L1/YKL40) had low discrimination ability and weak correlation with disease progression; however, CHI3L1 was independently associated with survival. A longitudinal study showed that CHIT1 and CHI3L1 were linked to the rate of disease progression and, consistent with previous findings, their levels remained stable over time [136]. These proteins are promising as prognostic and pharmacodynamic biomarkers [137].

### 8.3. Niclosamide

**Figure 1:**
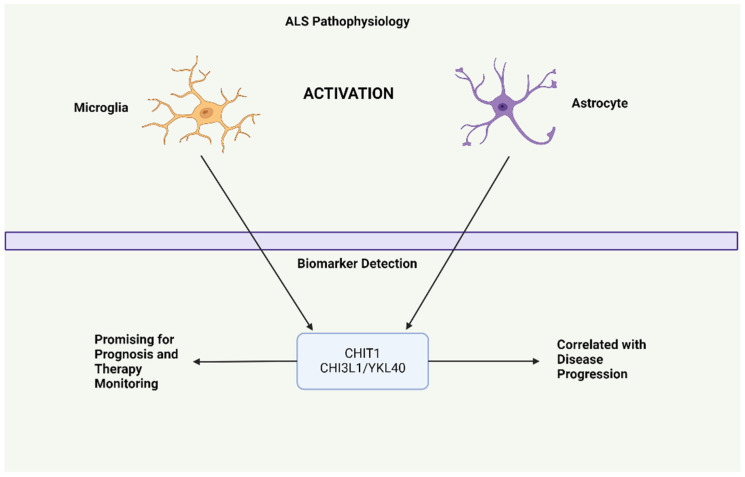
Role of Chitinase as a Biomarker in ALS Pathophysiology. This image illustrates how activated microglia and astrocytes in ALS contribute to chitinase expression, which serves as a biomarker for disease progression and therapeutic monitoring.

A recent study by Milani et al. (2024) explored the effects of Niclosamide on the *SOD1* gene mutation in two transgenic murine models, *SOD1*-G93A and *FUS* mice, with the drug administered intraperitoneally. Niclosamide belongs to the salicylanilide class of pharmacologic agents, characterized by an aryl β-hydroxy-carbonyl pharmacophore motif, commonly found in many biologically active natural products. Its primary action involves the translocation of protons across the mitochondrial membrane, leading to mild mitochondrial uncoupling. This mechanism is potent enough to kill tapeworms in the gastrointestinal tract while remaining generally well-tolerated by human cells. Milani et al. found that in vivo treatment with Niclosamide in 34 male and 20 female mice with the *SOD1* gene mutation, initiated at symptom onset and continued for approximately 160 days, and in 32 *FUS* mice treated for about 40 days, resulted in significant benefits. The probability of survival after treatment increased by 40%, and overall survival improved by approximately 20%. Additionally, Niclosamide delayed the onset of neuromuscular deficits and significantly enhanced muscular strength [138] (Figure 1).

## 9. Conclusions

In summary, this review has provided a detailed overview of amyotrophic lateral sclerosis (ALS), covering its epidemiology, pathophysiology, clinical presentation, diagnostic methods, and both current and emerging treatments. The key findings highlight the complexity of ALS, with both genetic and environmental factors playing important roles in its development. Advances in understanding molecular mechanisms like protein misfolding, mitochondrial dysfunction, oxidative stress, and axonal transport defects have helped clarify the disease’s progression and potential treatment targets.

These findings have significant implications for clinical practice. Improved diagnostic criteria and the identification of biomarkers can lead to earlier and more accurate diagnoses, potentially improving patient outcomes. Current drug treatments offer some benefits but highlight the need for more effective therapies. Emerging treatments, such as gene therapy and stem cell therapy, provide hope for changing the course of the disease and enhancing the quality of life for ALS patients.

Looking ahead, continued research is crucial to overcoming current challenges, such as the variability in disease presentation and the limited effectiveness of existing treatments. Further exploration of ALS’s molecular basis and the development of innovative therapeutic strategies are essential. Policymakers and healthcare providers must also consider these advancements, ensuring that new therapies are accessible to those in need and that healthcare systems support ongoing ALS research and patient care.

In conclusion, while there has been significant progress in understanding and treating ALS, much work remains. The future of ALS research holds promise for more effective treatments and, ultimately, a cure for this devastating disease. Collaborative efforts in research, clinical practice, and policy will be vital in making these advancements a reality.

## Figures and Tables

**Table 1:** Summary table of the genes described.

Gene	Aspect	Details
***C9ORF72***	Hexanucleotide Repeat Expansion	Located in the first intron of the *C9orf72* gene
***C9ORF72***	Pathogenic Repeat Size	Unknown, but a cutoff of 30 repeats is used in studies
***C9ORF72***	Healthy Individuals Repeat Size	Typically fewer than 11 repeats
***C9ORF72***	Patient Repeat Size	Several hundreds to thousands, some with 45−80 repeats
***C9ORF72***	Repeat Expansion Contribution in ALS	40% of familial ALS (FALS), 8% of sporadic ALS (SALS)
***C9ORF72***	Repeat Expansion Contribution in FTD	30% of familial FTD (FFTD)
***C9ORF72***	Cell Type Expression Distribution	High in myeloid cells, lower in lymphoid cells and other tissues
***C9ORF72***	Primary Organs of Expression	Mainly in brain, spinal cord, immune system; lower in lungs, heart, liver, kidney, skeletal muscle
***C9ORF72***	mRNA Isoforms	Three isoforms (V1−V3)
***C9ORF72***	Proteins Encoded	C9ORF72-long (C9-L), C9ORF72-short (C9-S)
***C9ORF72***	Abundant Isoform	C9-L (481 amino acids)
***C9ORF72***	Protein Localization	Predominantly cytoplasmic with punctate staining in neurites
***C9ORF72***	Function in Stress Granules	Involved in formation and degradation
***C9ORF72***	Protein Interaction (SMCR8)	Interacts through its DENN domain, involved in membrane trafficking and autophagy
***C9ORF72***	Implication in Neurodegenerative Disorders	Linked to ALS, FTD, and potential other neurodegenerative disorders
***TDP-43***	RNA Metabolism and Aggregation	RNA-binding, mislocalization, aggregation, and neurotoxicity in ALS
***SOD1***	Oxidative Stress and Mitochondrial Dysfunction	Mutations lead to oxidative stress, protein misfolding, and mitochondrial dysfunction in ALS
***FUS***	Nucleocytoplasmic Transport and Aggregation	Mutations disrupt RNA metabolism, protein mislocalization, and aggregation
